# A transcranial magnetic stimulation study on the role of the right angular gyrus in orienting and reorienting of attention toward threat

**DOI:** 10.3758/s13415-025-01275-3

**Published:** 2025-03-26

**Authors:** M. Lojowska, J. M. Gerbracht, J. B. Engelmann, K. Roelofs, M. Mulckhuyse

**Affiliations:** 1 Social Psychology, Leiden University, Leiden, The Netherlands; 2 Cognitive Psychology, Leiden University, Leiden, The Netherlands; 3 Center for Research in Experimental Economics and Political Decision Making (CREED), Amsterdam School of Economics, University of Amsterdam, Amsterdam, The Netherlands; 4 Amsterdam Brain and Cognition, University of Amsterdam, Amsterdam, The Netherlands; 5 Donders Institute for Brain Cognition and Behaviour: Centre for Cognitive Neuroimaging, Nijmegen, The Netherlands; 6 Behavioural Science Institute, Radboud University, Nijmegen, The Netherlands; 7 Wassenaarseweg 52, 2333 AK Leiden, The Netherlands

**Keywords:** Reorienting of spatial attention, Emotion, Angular gyrus, Transcranial magnetic stimulation

## Abstract

Reorientation of attention to threatening stimuli is a fundamental part of human cognition. Such interaction between cognitive and affective processes is often associated with faster response times. In the present study, the role of the right angular gyrus (AG) in reorienting to threat is examined. An exogenous spatial cueing paradigm was adopted with threatening and nonthreatening targets. Threat was induced by means of differential fear conditioning of the target. Single pulse transcranial magnetic stimulation (TMS) was applied to the right AG at different stimulus onset asynchronies (SOA) after target onset (range 30–300 ms). Transcranial magnetic stimulation was predicted to interfere at an earlier SOA with reorienting (during invalidly cued trials) to threatening targets. Even though an overall decrement in performance to targets contralateral to TMS stimulation was found, TMS to right AG did not specifically affect reorienting, neither to safe nor to threatening targets. We suggest that detection of biologically significant stimuli outside the focus of attention may depend more on the ventral frontoparietal rather than dorsal frontoparietal network of attention.

**Supplementary Information:**

The online version contains supplementary material available at 10.3758/s13415-025-01275-3.

## Introduction

Goal-directed attention allows us to engage purposefully with and act upon our environment (Driver, 2001). Stimulus-driven attention permits important flexibility to perceive and engage with salient stimuli (Corbetta et al., 2008; Theeuwes, 2010). Shifting visual spatial attention is generally associated with dorsoparietal regions (Corbetta & Shulman, 2002). Specifically, the right posterior parietal cortex (PPC) has been associated with directing attention toward salient stimuli (Hodsoll et al., 2009). Said salient stimuli may require immediate attention in order to adaptively respond to threat.

It is suggested that visual selection of threatening stimuli is mediated by a fast, subcortical, magnocellular pathway bypassing early visual cortex (Bocanegra & Zeelenberg, 2009; Lojowska et al., 2015). Several authors argue that such a phylogenetically older, retinotectal pathway interacts with cortical networks and modulates visual perception and attention (LeDoux, 2000; Tamietto & De Gelder, 2010; Vuilleumier, 2005). Indeed, emotionally salient stimuli appear to elicit earlier neural responses (Carretié et al., 2004; Pourtois et al., 2010) and seem prioritized in visual search (Öhman et al., 2001; Mulckhuyse & Dalmaijer, 2015; Notebaert et al., 2011; Schmidt et al., 2015; Yiend, 2010).

To gain more precise understanding of the role of the PPC when orienting to emotionally relevant stimuli, we used an adaptation of a single-pulse TMS study by Chambers et al. (2004) in which the time-course of reorienting to behaviorally relevant stimuli (targets) was investigated in a spatial cueing task. Chambers et al. applied single-pulse TMS individually and unilaterally to the supramarginal gyrus (SMG) and the angular gyrus (AG) after target onset. No effects were found for validly cued trials where no reorientation was required. In contrast, for invalidly cued trials, a significantly decreased response accuracy was observed. This decrease occurred in a biphasic pattern during 90–120 ms and 210–240 ms after target onset (SOA) and was noted for the right AG only. The authors hypothesized that both fast and slower visual pathways may be distinctly relevant to the observed response pattern. As such, Chambers et al. (2004) suggested a retinotectal and a geniculostriate pathway as potential explanation and underlying cause for the observed biphasic involvement of the right AG in reorientation of attention.

In the present study, involvement of the right AG was closer evaluated under the premise of a threatening target. Assuming prioritization of threatening stimuli occurs via a retinotectal pathway, faster processing is anticipated for threatening targets compared to nonthreatening targets. Therefore, reorienting to an emotionally relevant target is expected to be mediated by the right AG at an earlier time window than reorienting to a nonemotionally relevant target. Moreover, high anxious individuals demonstrate faster orienting, especially to briefly presented highly aversive stimuli (Cisler & Koster, 2010). However, to our knowledge, this has never been investigated in the context of reorienting to threat in a spatial cueing task.

To preserve comparability, the experimental setup and methodology of Chambers et al. (2004) was largely followed, although we adopted a slightly different discrimination task and did not measure eye movements (see supplement Chambers et al., 2004). Moreover, we used a larger sample size (N = 3 in Chambers et al. (2004) vs. N = 22 in the current study). As such, a spatial cueing task and single-pulse TMS was used with healthy subjects. To introduce a threatening stimulus, differential fear conditioning was applied. Similar to Chambers et al. (2004), we expected no effect of TMS for the validly cued trials, but a TMS modulation for the invalidly cued trials. That is, we expected an earlier decrease in response accuracy to invalidly cued threatening targets compared to safe targets.

## Materials and methods

### Participants and ethics

The present sample size was oriented on findings by Chambers et al. (2004) who reported significant effects with ~ 230 observations per data point given by three participants. A total of 22 right-handed volunteers, 9 males and 13 females, aged 19 to 26 years, participated with a mean STAI score of 36.23 (SD = 9.26; Table 1).

We conducted a sensitivity analysis for the interaction between cue, threat, and TMS-SOA. We used simr package (Green & Macleod, 2016) in R (R Core Team, 2021) to determine the power of detecting the interaction between the cue validity, threat condition, and TMS-SOA in our model with 22 participants. For the analysis, we used the existing effect sizes from the actual model (Table S1). The analysis revealed that the interaction between these three factors with the provided effect sizes was possible to detect with 80% power and alpha of 0.05 with 17 participants (Fig. S2). The script used for this analysis can be found here: https://osf.io/gbyhj/.

All subjects were screened for contraindications to noninvasive brain stimulation (Keel et al., 2001), history of psychiatric or neurological disease, color blindness, and vision problems. Consequently, all subjects were healthy and had normal or corrected-to-normal vision. Prior to the investigation, informed consent was obtained in writing, whereas research objectives remained unknown to the subjects. The study was approved by the *Medical Ethics Committee of the Radboud University Medical Center Nijmegen* (NL52504.091.15 CMO Region Arnhem- Nijmegen, The Netherlands). Transcranial magnetic stimulation parameters were in agreement with the *International Federation of Clinical Neurophysiology* safety guidelines (Rossi et al., 2009) and in accordance with the standards set by the *Declaration of Helsinki*.

### Apparatus and stimuli

The experimental task was administered using *E-prime 2.0* and responses were recorded via the *Serial Response Box* (Psychology Software Tools, Sharpsburg, Inc., PA). Stimuli were displayed using a *BENQ XL 2420 T* monitor with a refresh rate of 100 Hz. A light grey fixation cross (0.5 cm) was presented in the centre of a dark grey background. The cue consisted of a white filled circle (0.8 cm) presented 8.5 cm to either the left or the right of fixation. The target stimulus consisted of a square (1.2 cm * 1.2 cm) made up of 11 blue or yellow line elements, which were presented 8.5 cm to either the left or the right of fixation. The line elements were matched for luminance $\left(39\frac{cd}{{m}^{2}}\right)$ and tilted 45° to either the right or to the left side. The mask was a random pattern of black line elements (1.5 cm*1.5 cm) presented at the location of the target stimulus.

Shocks were applied by using a *Digitimer Constant Current Stimulator DS7A* (www.digitimer.com) and standard Ag/AgCl electrodes attached to the fourth and fifth phalanges of the left hand. The maximum intensity stimulus consisted of 10 pulses of 1 ms length and 19.75 ms ISI, administered during a 200-ms time interval at 50 Hz with a maximum intensity of 6 mA.

The right AG was localized using scalp coordination marked on T1-weighted MRI scans and *Brainsight* (Rogue Research Inc., Montreal, Quebec, Canada) neuronavigation. Here, the right AG was defined as the region directly adjacent to the dorsolateral projection of the superior temporal sulcus, which bifurcates the AG (Chambers et al., 2004). At the target area, the TMS coil was placed tangential to the scalp with the handle pointing caudally in a fixating custom coil holder. Transcranial magnetic stimulation was delivered with a biphasic pulse configuration using a *MC-B65-HO* figure-of-8 coil (MagVenture, Farum, Denmark) connected to a *Magpro-X-100* magnetic stimulator (MagVenture, Farum, Denmark).

To measure trait anxiety, participants completed The Trait Anxiety Inventory Questionnaire (STAI-T; Spielberger et al., 1970; Van der Ploeg, 1980). Subjective ratings were obtained using Likert scales (ranging from 1, “not at all,” to 9, “extremely”). To measure subjective fear towards the CS + and CS − stimuli, participants were asked to indicate the extent to which they felt anxious or fearful when they saw the blue (yellow) lines. To measure contingency awareness (i.e., the association between the CS + stimuli with a chance of receiving an electric shock), participants were asked to what extent they expected to receive a shock when the blue (yellow) lines were presented. In addition, participants were asked to judge the intensity, unpleasantness and fear of the US stimulus (shocks) also using a Likert scale.

### Design

The spatial cueing task consisted of twenty blocks of 44 trials that were divided over two separate sessions due to the length of the experiment. The sessions were on different days with at least 1 week in between. In half of the trials the CS + and the other half the CS − target was presented. The cue was 50% valid. Single-pulse TMS was applied at one of ten different intervals following target onset, or no TMS was applied. These ten stimulus-onset asynchronies (SOA) were 30–300-ms long (30-ms increments). All trials were presented randomly within a block. In each block, two CS + trials were additionally reinforced with a shock 800 ms after target onset to avoid extinction (Mackintosh, 1983). Here, no TMS pulse was applied. Said reinforcement trials were excluded from analyses. Hence, a total of 880 (440 CS − targets and 440 CS + targets) experimental trials were considered in final analyses.

### Procedure

In the first session, resting motor threshold (MT) was determined for the right hemisphere using the thumb movement visualization method after single pulse TMS (Schutter & van Honk, 2006). Subsequently, TMS output was set at 110% and lowered in case of muscular artefacts. The resulting TMS output and the following procedure were used in both sessions. The experiment started with a practice session of 32 trials. Here, a mock-up TMS (upwards pointing coil) was used. Participants with at least 85% accuracy in practice trials were permitted to begin the next phase; otherwise, they had to repeat the practice session.

Subsequently, electrodes were attached and intensity of shock output was determined with a calibration procedure. A standardized staircase procedure comprising five shocks (Cornsweet, 1962) was used. The participants reported shock unpleasantness verbally on a scale from 1–5 where 1 corresponds with “not at all” and 5 with “very much.” For each subject, the level of shock unpleasantness was set at 4.

A fear conditioning acquisition phase was conducted prior to the experiment, which consisted of 16 trials, in which the yellow (8 trials) or blue target stimulus (8 trials) was presented for 1000 ms. Participants were asked to observe but did not have to respond to the target stimulus. One of the colors was paired with a shock (CS +) at stimulus offset, whereas the other color was not (CS −). Reinforced colors were consistent for each participant between sessions and counterbalanced between subjects. After the acquisition phase, participants were asked to indicate which color was linked to the shock. Correct identification of the conditioned stimulus was necessary to continue the study.

**Fig. 1:**
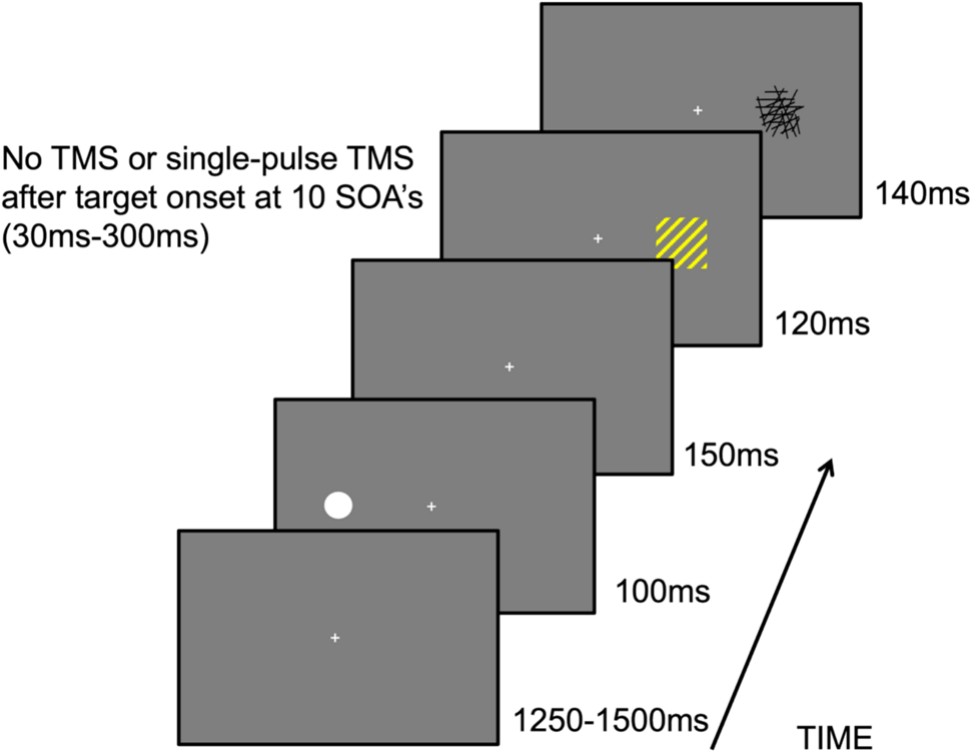
Threat-based exogenous spatial cueing task – example of an invalid trial. *Note*. Participants had to indicate as quickly and accurately as possible whether the orientation of the line-elements, yellow or blue, were orientated to the left or right. Preceding the target, a cue—white circle—was presented either on the same (valid trials) or the opposite side of the target (invalid trials). The color of the target was conditioned to shocks, thereby signaling a threat or safety. The TMS pulse was administered at 10 different SOAs following the target presentation

Each experimental trial began with 1250-ms presentation of a fixation cross and additional jitter between 50 and 250 ms (Fig. 1). Subsequently, the cue was presented for 100 ms and appeared randomly in equal distribution (50%) either left or right from the fixation cross. After a delay of 150 ms, the target was presented for 120 ms followed by a random pattern mask of black lines for 140 ms. Participants were instructed to identify the orientation of the line-elements as either “left” or “right” by pressing “1” with their right index finger for left orientation and “2” with their right middle finger for right orientation. Each trial ended when a response was given, i.e., there was no constraint for the response time.

After each block of 44 trials, participants received feedback about their response accuracy. Here, breaks were allowed, and the position of the coil was inspected and readjusted if needed. After completion of each session, contingency and fear ratings for CS + and CS − as well as ratings of intensity, unpleasantness, and fear of the US were obtained. At the end of session 2, participants filled out the English or Dutch version of the trait anxiety inventory questionnaire (STAI-T; Spielberger et al., 1970; Van der Ploeg, 1980).

### Statistical analysis

Statistical analyses were carried out in R (Version 3.5.1; R Core team, 2016). To preserve comparability with Chambers et al. (2004) and owing to our online TMS protocol with different target-TMS SOAs, which may affect reaction times (Duecker et al., 2013), our main analyses were focused on accuracy. Accuracy was coded as a binary variable for which logistic mixed-effects models were implemented via the glmer function (lmer4 package, version: 3.3.1, Bates et al., 2015). Each model included fixed effects for cue validity (valid and invalid condition), threat condition (CS + , CS −), and TMS condition (present vs. absence of TMS; or TMS stimulus onset asynchrony [SOAs]), as well as the interactions between cue validity, threat condition, and TMS. All models included random intercepts for each subject. Session number (1,2) was used as a covariate. We included STAI in the model to examine whether individual differences in trait anxiety can explain the behavioral responses. We also examined the effects of TMS on RT, and whether RT can explain accuracy (RT-accuracy tradeoff).

Owing to the complexity of our models, we did not employ a maximal random-effects structure (Barr et al., 2013), but report results from a simpler random intercepts only model for all analyses. In Wilkinson-Rogers notation, the GLMEs models for accuracy analysis writes as follows:

Correct = Cue validity * Threat condition * TMS (TMS-SOAs) + Session + (1| Subject).

As a general strategy, we first used an omnibus model investigating the main effects of cue validity, threat condition, TMS, and their interactions. To this end, *p*-values were determined using Type 3 Likelihood ratio tests implemented in the *mixed* function of the package *afex* (Singmann et al., 2015, 2021, 2023). In logistic mixed-effect models, odds ratio and their 95% CI were calculated as a measure of significance of the effects. In linear mixed effects models with RT as dependent variable, point estimates (B) were used as a measure of the magnitude of the effects using *lmerTest* package (Kuznetsova et al., 2017). In the model testing the influence of RT on accuracy, we used a log of RT in the analysis to correct for its skewed distribution. In all models, continuous predictors (ratings of STAI-T and log RT) were centered, and all categorical predictors were coded using sum coding.

In addition to our main analysis, we conducted several exploratory analyses on the same data sets to get a better understanding of the effects observed in the main analysis. To address this multiple testing, we have applied a Bonferroni correction to the nested models. For the models assessing the perception of UC (intensity, unpleasantness and fear), the significance threshold was adjusted to α = 0.05/6 = 0.0083 (because both one-sample *t*-test and ANOVA was used for each measure). Regarding the main analysis on the effect of TMS-SOA on accuracy (Table S1), we performed additional exploratory analyses that included STAI (Table S2), target position (Table S4), the main effect of TMS (present vs. absent, Table S6), and RT (Table S8), and trial number (Table S10) as (additional) predictors. This resulted in seven models for accuracy, for which the significance threshold was adjusted to α = 0.05/7 = 0.0071. Models reported in Tables S3, S5, and S9 were considered robustness checks or follow-up analyses and were not included in the calculation of the adjusted α.

Trials on which shocks were delivered were excluded from the final analysis. We also excluded trials in which participants gave too fast or too slow responses (faster than 350 ms, and slower than 3000 ms; 1.40% of all trials). These cutoffs were used to ensure that the trials used in the analysis included trials for which a response was given following the TMS pulse (max. SOA-TMS was 300 ms; therefore, the cutoff for TMS trials was set to 350 ms, 17.61% of all trials). Subsequently, we excluded RT outliers at individual level based on ± 2.5 SD criterion from the mean (2.18% of all trials). The final data set included 15,268 trials, comprising of 7636 CS + trials and 7632 CS − trials. See Table S11 in the Supplementary material, summarizing the final number of trials for each condition.

Furthermore, we excluded trials for which there were large timing inaccuracies with regard to the timing of the TMS pulse from final analyses. The timing of the TMS pulse for each SOA condition showed high variances with standard deviations between $3.78$ ms and $11.20$ ms. Based on bimodal SOA distributions (see Supplementary material, Fig. S1), a 4-ms cutoff criterion above the expected timing of the TMS pulse was chosen, and trials falling within this criterion were included in the analysis (e.g., for a TMA SOA of 30 ms, trials in which a TMS pulse was delivered until 34 ms were included in the analysis). Following this criterion, 17.86% of the trials were excluded from the analysis. An analysis including trials showing high TMS-SOA variability yielded similar results (Supplementary Materials Table S9).

## Results

### Manipulation check: Fear conditioning

Fear conditioning was successful both in terms of subjective fear and contingency awareness. We first examined participants’ ratings of fear and contingency for the CS + and CS − stimuli, and whether these scores differed between the two sessions. A main effect of threat condition on fear rating *F*(1,21) = 33.23, *p* < 0.001, showed that the participants experienced more fear in response to the CS + stimulus (M = 3.57, SD = 1.73) than the CS − stimulus (M = 1.32, SD = 0.75, Table 1). A main effect of threat condition on contingency ratings *F*(1,21) = 97.63, *p* < 0.001, indicated that the participants expected to receive shocks more during the CS + (M = 4.16, SD = 1.29) than during the CS − stimuli (M = 1.14, SD = 0.35). Importantly, the interaction between threat condition and session number was non-significant for the ratings of fear *F*(1,21) = 3.32, *p* = 0.083, and contingency *F*(1,21) = 0.27, *p* = 0.608, suggesting that these ratings did not differ between the two sessions.

**Table 1:** Descriptives of ratings for the fear conditioning and STAI-T scores

	Fear		Contingency		US (shocks)			STAI-T
	CS +	CS −	CS +	CS −	Fear	Intensity	Unpleasantness	
Min	1	1	1	1	1	1.5	1	23
Max	6.5	4	6.5	2.5	7.5	7	8	55
Mean	3.57	1.32	4.16	1.14	3.89	4.61	4.41	36.23
SD	1.73	0.75	1.29	0.35	1.92	1.53	1.74	9.26

*STAI-T* State and Trait Anxiety Inventory – Trait, *US* unconditioned stimulus

Next, we verified whether the ratings of the US (shocks) were significantly higher than the minimum possible rating, (i.e., 1). One-sample *t*-tests confirmed that this was the case for shock intensity (M = 4.61, SD = 1.53, *t*(21) = 9.21, *p* < 0.001), unpleasantness (M = 4.41, SD = 1.74; *t*(21) = 11.04, *p* < 0.001), and “fear of receiving a shock” (M = 3.89, SD = 1.82; *t*(21) = 7.05, *p* < 0.001). Importantly, the US ratings did not differ between the sessions, as indicated by a nonsignificant interaction between the session and threat condition for the ratings of intensity *F*(1,21) = 0.15, *p* = 0.706, unpleasantness *F*(1,21) = 0.88, *p* = 0.358, and fear *F*(1,21) = 0.20, *p* = 0.658. Overall, these results suggest that fear conditioning was successful in our study. After applying the Bonferroni correction for multiple comparisons (α adjusted = 0.05/6 tests = 0.0083), the results from one-sample *t*-tests remained significant.

### No time-specific interference of attentional orienting or reorienting

**Fig. 2:**
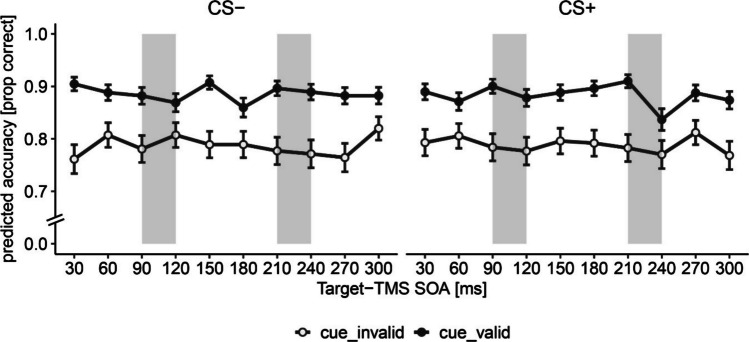
Accuracy for safe (CS −) and threat (CS +) targets followed by a TMS pulse at different SOAs. *Note*. The grey areas indicate the time windows of interests (TOIs), in which the effect of TMS applied to the right AG was previously found to reduce accuracy in invalid trials (Chamber et al., 2004). Error bars represent standard errors of the mean. SOA = stimulus-onset asynchrony; prop = proportion; ms = milliseconds

First, we focused on the time delineated effects of TMS per SOA. To identify the effects of TMS-SOA, our analysis involved a full interaction model with the following predictors: TMS-SOA, cue validity and threat condition, and accuracy as the dependent variable. Session number (1,2) was used as a covariate. We found a significant main effect of cue validity, where the odds of correct responses were significantly larger in valid than in invalid trials, indicating that the peripheral cue captured attention: OR = 0.67, 95% CI [0.64, 0.71], χ^2^(1) = 252.68, *p* < 0.001 (Fig. 2). We also found a main effect of session on accuracy, indicating that participants performance improved with time: OR = 1.44, 95% CI [1.37, 1.51], χ^2^(1) = 49.37, *p* < 0.001. These results remain significant after applying the Bonferroni correction. The interactions between the cue validity and TMS-SOA, and between cue validity, threat condition, and TMS-SOA were nonsignificant (*p* > 0.05; full model results are provided in the supplementary materials Table S1). Including STAI-T as a predictor did not change the results (Table S2). There was also no significant influence of time (trial number) on the effects of TMS-SOA and threat condition, also not as a function of cue validity, on accuracy (Table S10).

### No interference of reorienting to invalid trials during TOIs: 90–120 ms and 210–240 ms

Following the findings of Chambers et al. (2004) in which a TMS-induced decrease in performance accuracy was observed for invalid trials at the time-windows between 90 to 120 ms and between 210 to 240 ms, we performed an exploratory analysis focusing specifically on these SOAs. For each of these time-windows, we estimated a model, including accuracy for the four TMS-SOAs within the time-windows of interest (for the first time-window, we used the following TMS-SOAs: 60 ms 90 ms, 120 ms, 150 ms; for the second time-window, we used the following TMS-SOAs: 190 ms, 210 ms, 240 ms, and 270 ms). None of these comparisons yielded a significant interaction effect between the cue validity and TMS-SOA, as well as between cue validity, threat condition, and TMS-SOA (*p* > 0.05; Table S3).

### TMS induced interference of target processing in contralateral visual field, irrespective of threat and cue-validity

**Fig. 3:**
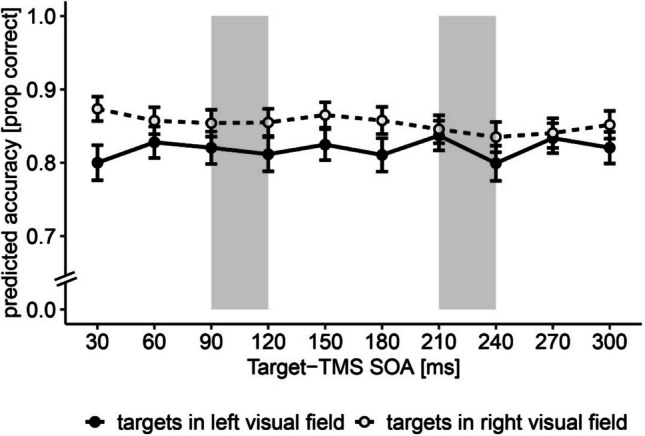
Accuracy for targets presented in the ipsilateral (open circles) and contralateral (filled circles) visual field. *Note*. Gray areas indicate the time windows of interests (TOIs), in which the effect of TMS applied to the right AG was previously found to reduce accuracy in invalid trials (Chamber et al., 2004). The results demonstrate accuracy for TMS pulse applied over the right AG for the targets presented in the left and right visual fields. Error bars represent standard errors of the mean. Prop = proportion

Because laterality effects are a common finding in TMS studies (Bourgeois et al., 2013; see for review Duecker & Sack, 2015), we probed our data for laterality effects and tested whether the SOA–validity interaction is dependent on visual field by adding target location to our model. There was a significant main effect of the location of the target on accuracy, with higher accuracy for targets presented in the right (M = 0.85, SD = 0.36) than the left visual field (M = 0.82, SD = 0.39), indicating a typical laterality effect of TMS in the contralateral visual field OR = 0.87, 95% CI [0.83, 0.91], χ^2^(1) = 29.37, *p* < 0.001 (Fig. 3). The interaction between TMS-SOA and target location was nonsignificant, χ^2^(9) = 13.27, *p* = 0.151. For full model results, also after applying the Bonferroni correction, see Table S4). An analysis specifically focusing on targets in the left visual field in the invalid trials did not yield any significant effects (*p* > 0.05; Table S5).

### TMS-induced interference of threat processing

**Fig. 4:**
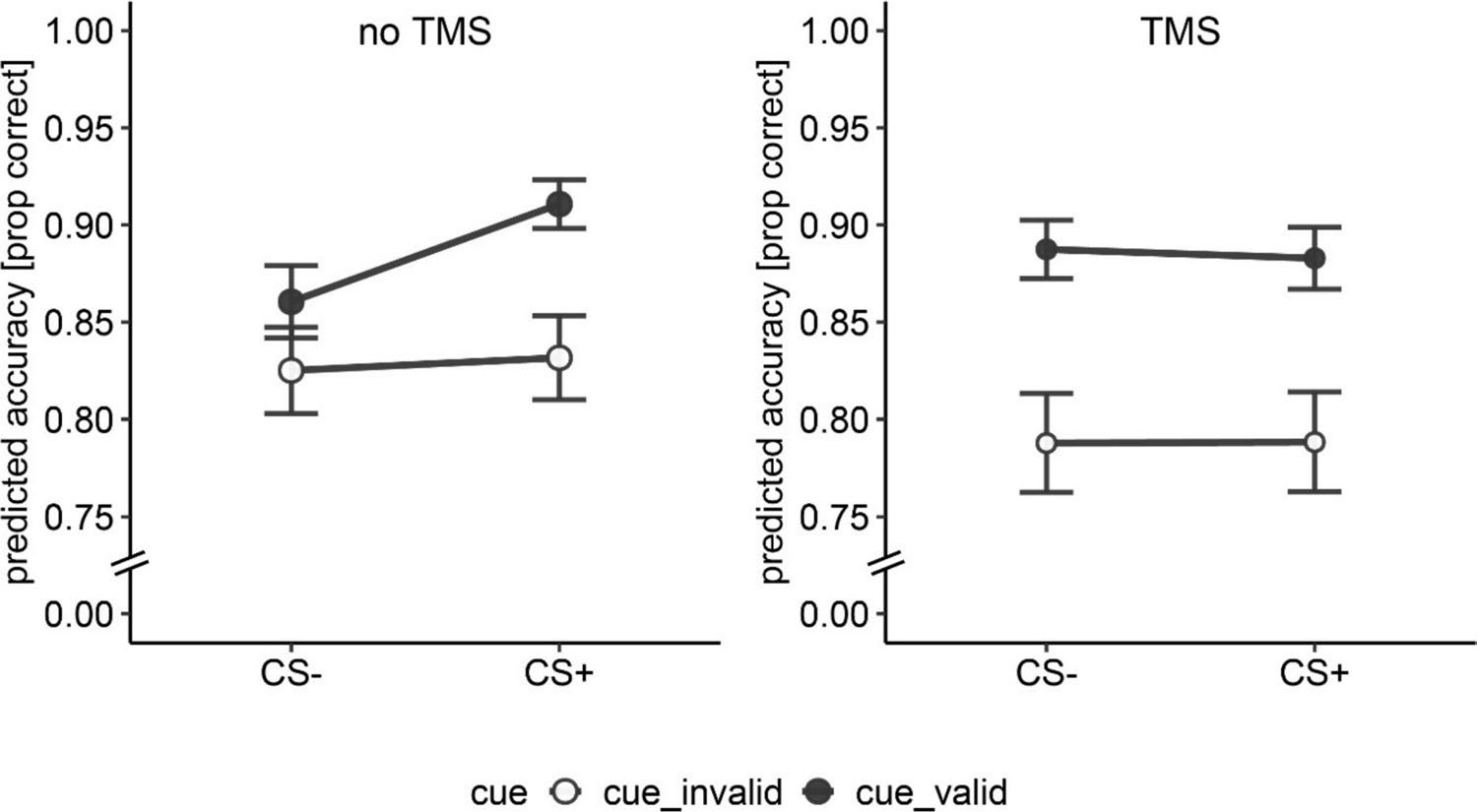
Accuracy in the spatial cueing task in the absence and presence of TMS (across all SOA’s) for safe (CS −) and threat (CS +) targets. Note. Values represent fitted values from the glmer model. Error bars represent standard errors of the mean. prop = proportion

To determine the overall effect of TMS on orienting and reorienting toward safe and threat targets, we collapsed across all SOAs, testing the effects of TMS, cue validity, and threat on accuracy. Similar to the full model that included TMS-SOA, we found a main effect of cue validity, OR = 0.72, 95% CI [0.67, 0.78], χ^2^(1) = 73.52, *p* < 0.001 (Fig. 4), and a main effect of session, OR = 1.41, 95% CI [1.29, 1.55], χ^2^(1) = 51.19, *p* < 0.001. In addition, we found a main effect of TMS, indicating better accuracy during no-TMS compared with TMS stimulation OR = 1.09, 95% CI [1.00, 1.17], χ^2^(1) = 4.76, *p* = 0.029. The interaction between TMS and threat was marginally significant, χ^2^(1) = 3.66, *p* = 0.055. However, this result was nonsignificant after applying the Bonferroni correction (α = 0.0071). The remaining interactions were nonsignificant (*p* > 0.05). Full model results are provided in Supplementary Material Table S6.

**Fig. 5:**
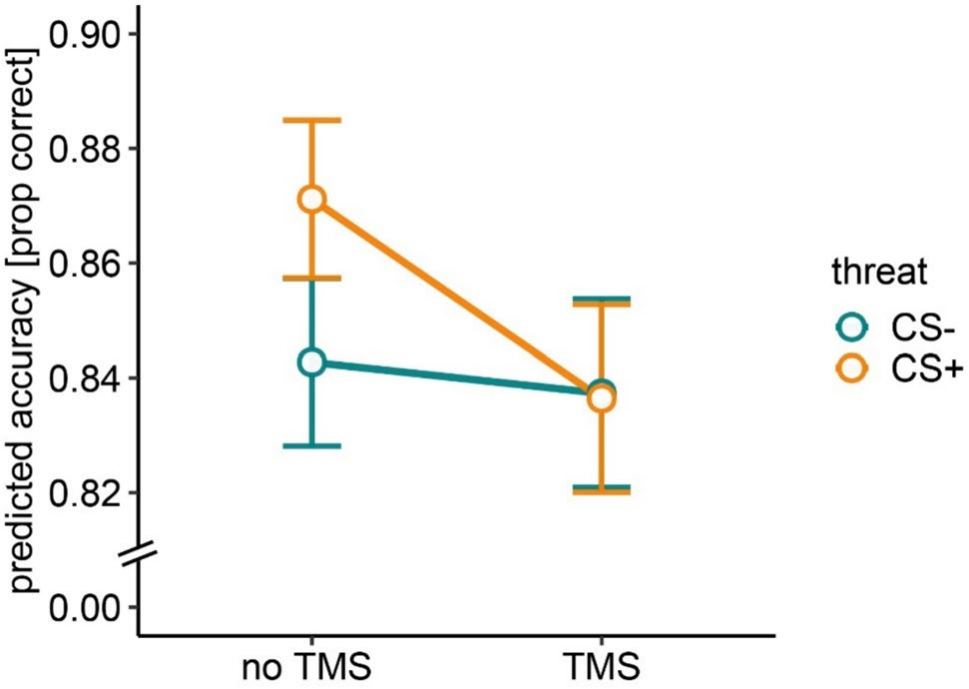
Accuracy in the spatial cueing task in the absence and presence of TMS for safe (CS −) and threat (CS +) targets. Values represent fitted values from the glmer model. Error bars represent standard errors of the mean. prop = proportion

We nonetheless explored the interaction between threat and TMS condition, and this revealed a significant difference in accuracy between CS + and CS − condition in the no-TMS condition (Fig. 5). Specifically, in the no-TMS condition, the participants showed marginally significant higher accuracy during threat (M = 0.87, SD = 0.33) than during safe trials (M = 0.84, SD = 0.36): OR = 0.86, 95% CI [0.75, 0.99], χ^2^(1) = 3.71, *p* = 0.053. The difference between threat and safe trials in TMS condition was nonsignificant; OR = 1.00, 95% CI [0.96, 1.05], χ^2^(1) = 0.04, *p* = 0.838.

### No Speed-accuracy Trade-offs

Similar to the analysis on accuracy, the analysis on RT revealed a main effect of cue validity with faster responses on valid (M = 562.98 ms, SD = 78.74) compared with invalid trials (M = 589.95 ms, SD = 78.74), confirming the typical cue-validity effect, *B* = 14.00, SE = 1.70, χ^2^(1) = 68.93, *p* < 0.001. However, none of the other effects were significant (*p* > 0.05; Table S7), nor did we find evidence of speed-accuracy trade-off (*p* > 0.05, Table S8).

## Discussion

The present study elaborates on findings by Chambers et al. (2004) who reported interference with attentional reorienting during invalid trials in an exogenous spatial cueing task at two time-windows (90–120 ms and 210–240 ms) when TMS was applied to the right AG. The objective of the present study was to further investigate the nature and time course of right-AG involvement when reorienting attention toward *threatening* stimuli. Extending the well-established spatial cueing paradigm, a fear conditioned target was introduced to allow for an evaluation of emotional response patterns. The fear conditioned target was expected to induce prioritized processing and hence earlier reorienting to the invalidly cued threatening target. Therefore, we expected TMS to interfere with performance to invalidly cued threatening targets at earlier time-windows compared with safe targets.

Subjective ratings indicated that fear-conditioning was successful. Participants were aware of the contingency between the color and the shock and were more afraid when the CS + was presented relative to the CS − . Furthermore, in the No-TMS condition, the cueing effect was stronger for the CS + target trials than the CS − target trials, which was driven by better performance on the valid CS + trials. This indicates enhanced orienting to threatening information (Koster et al., 2004).

During the TMS trials, an overall cueing effect was consistently observed, confirming a large body of previous work (Posner, 1980). Performance was improved in valid compared with invalid trials. This finding demonstrates that the cueing paradigm was effective. Specifically, the peripheral cue captured attention and facilitated performance for targets at the attended location (valid trials) and impaired performance for targets at the unattended location (invalid trials). However, no effect of TMS was observed during reorienting, neither for threat nor for safe trials. If we assume that attention had to be reoriented from the cue to the opposite target during invalid trials, our findings do not provide any evidence for the mediation of the right AG in reorienting in the presence of both nonthreatening and threatening stimuli, which is inconsistent with the findings reported by Chambers et al. (2004).

It is important to note that in the current task re-orienting was induced by a salient but at the same time task relevant stimulus. In the model by Corbetta and Shulman (2002), a right lateralized ventral frontal network is activated when a task relevant stimulus is detected outside the focus of attention. This network consists of the temporoparietal junction (TPJ) and ventral frontal cortex (VFC), in which the rTPJ is supposed to act as a circuit breaker of ongoing task sets (Doricchi et al., 2009). Indeed, previous TMS studies showed that in addition to parietal regions, the rTPJ is involved in exogenous spatial attention (Bourgeois et al., 2013; Chica et al., 2011). The role of the ventral network as a circuit breaker is especially highlighted in a study by Chang et al. (2013), in which TMS to rTPJ modulated reorienting to task relevant distractors, whereas stimulating rFEF did not (Chang et al., 2013). In the current study, the target stimulus was not only task-relevant (as in Chambers et al. (2004)) but also biologically significant, as it signaled either threat or safety. Therefore, we may not compare our CS − target with a neutral target, because it had gained significant biological valence. Detection of biologically significant stimuli outside the focus of attention may rely more on areas in the ventral network, such as rTPJ (Corbetta & Shulman, 2002) than dorsal network, such as rPPC (but see Mulckhuyse et al., 2017). Subsequently, the act of voluntarily reorienting toward the target was mediated in conjunction with the *bilateral* frontoparietal network (Vossel et al., 2014). If indeed reorienting was mediated by the bilateral frontoparietal network, unilateral interference of the right AG may then affect reorienting less. For example, in a study that used an Attentional Network Task (ANT) in which right PPC was inhibited with continuous theta-burst stimulation (cBTS) no effect was found on orienting, while effects on alerting and executive functioning did emerge (Middag-van Spanje et al., 2022). The authors attributed the lack of an effect on orienting to the unilateralized stimulation, because voluntarily orienting of attention is mediated by the bilateral frontoparietal network. If reorienting in our study was mediated by the bilateral frontoparietal network, a single pulse to right AG might not have sufficed to interfere with reorienting. Alternatively, stimulation output of the single pulse was too low. In Chambers et al. (2004) ~ 110% of phosphene threshold was used, which in general requires a higher stimulation output (Phylactou et al., 2024) than the motor threshold used in the current study. Indeed, most TMS studies on spatial attention used a train of pulses (Heinen et al., 2011; Silvanto et al., 2009; Taylor et al., 2011, see for review Seghier, 2023), which might be necessary to affect reorienting processes. However, a recent study using online repetitive TMS and offline inhibitory stimulation of left IPS on temporal orienting also found no evidence (Capizzi et al., 2023). Similar to spatial orienting, the network involved in temporal orienting is widespread. Capizzi et al. tentatively suggested that interference of a specific node in a widespread network may be compensated for by activity in these other brain areas. This may also explain why we did not find impaired reorienting in our study.

Even though our stimulation protocol may not have been effectively interfering with reorienting, we did find an effect of target location, irrespective of cue validity, and threat. Performance was decreased to targets presented contralateral to TMS stimulation, which is in accordance with previous TMS studies that stimulated rPPC in various spatial attention tasks (Bourgeois et al., 2013; see for review Duecker & Sack, 2015). An alternative explanation for this finding might be that the TMS stimulation to the right hemisphere induced a covert shift of attention to the ipsilateral visual field simply due to the clicking sound. Indeed, it has been shown that lateralized sham TMS increased performance to targets presented in the ipsilateral visual field (Duecker & Sack, 2013). However, this effect was shown with TMS stimulation prior to and not after target onset as in our experiment. Moreover, if indeed the clicking sound induced a shift of covert attention, we could have expected to find a main effect of visual field, with faster responses to targets in the right compared with the left visual field and in addition an interaction effect of visual field with TMS SOA on RT; with a linear increase in reaction time to targets presented in the right visual field depending on TMS SOA that should be absent or less pronounced in the left visual field. Because we did not find a main effect of Visual Field nor an interaction on reaction time (Table S12), our finding seems to corroborate studies showing the involvement of right AG in spatial attention. However, our findings do not provide evidence for a dominant right lateralized role in reorienting to emotional stimuli.

We conclude that single pulse TMS to right AG does not interfere with reorienting in an exogenous spatial cueing task to targets that signal threat or safety. Possibly, detection of biologically significant stimuli outside the focus of attention relies more on involvement of the right lateralized ventral frontoparietal network after which reorienting is mediated by the bilateral dorsal frontoparietal network. Future repetitive TMS studies could elucidate the role of the rTPJ in reorienting to biologically significant stimuli outside the focus of attention.

## Supplementary Information

Below is the link to the electronic supplementary material.

Supplementary file1 (DOCX 132 KB)

## Data Availability

All data are available at the Open Science Framework (OSF) and can be accessed at https://osf.io/gbyhj/. This study’s design and its analysis were not pre-registered.

## Code availability

Analyses code is available at the Open Science Framework (OSF) and can be accessed at https://osf.io/gbyhj/. Data were analyzed using R (Version 3.5.1; R Core team, 2016).
