# Supplementary material for: A transcranial magnetic stimulation study on the role of the right angular gyrus in orienting and reorienting of attention toward threat
Source: Cogn Affect Behav Neurosci. 2025 Mar 26;25(3):668–78. doi: 10.3758/s13415-025-01275-3 (PMC12130136; doi:10.3758/s13415-025-01275-3)
Supplement: Supplementary file 1 — Supplementary file1 (DOCX 132 KB) [file 13415_2025_1275_MOESM1_ESM.docx]

**Supplementary Material**

A transcranial magnetic stimulation study on the role of the right Angular Gyrus

in orienting and re-orienting of attention towards threat

Lojowska, M.^1*^, J.M. Gerbracht^2*^, J. B. Engelmann^3,4^, K. Roelofs^5,6^, M. Mulckhuyse^2,5^

^1^Social psychology, Leiden University Leiden, The Netherlands

^2^Cognitive psychology, Leiden University Leiden, The Netherlands

^3^ Center for Research in Experimental Economics and Political Decision Making, University of Amsterdam, the Netherlands

^4^Amsterdam Brain and Cognition, University of Amsterdam, the Netherlands

^5^Donders Institute for Brain Cognition and Behaviour: Centre for Cognitive Neuroimaging, Nijmegen, The Netherlands

^6^Behavioural Science Institute, Radboud University, Nijmegen, The Netherlands

*^*^* authors contributed equally to this article.

**Controlling for variance of TMS timing**

Since hardware limitations can introduce uncertainties to the exact timing of the TMS pulse, we first checked whether the pulse was indeed administered at the intended time point. Timing of the TMS pulse for each SOA condition showed high variances with standard deviations (SD) between $3.78$ ms and $11.20$ ms. Notably, SOA distributions are consistently bimodal, with a right tail. Consequently, following a 4 ms cut-off criterion 17.82 % of trials were removed.

**Figure S1.** SOA values and the actual distribution of TMS pulses. Cut-off criterion (4ms) in

red. SOA, stimulus onset asynchrony


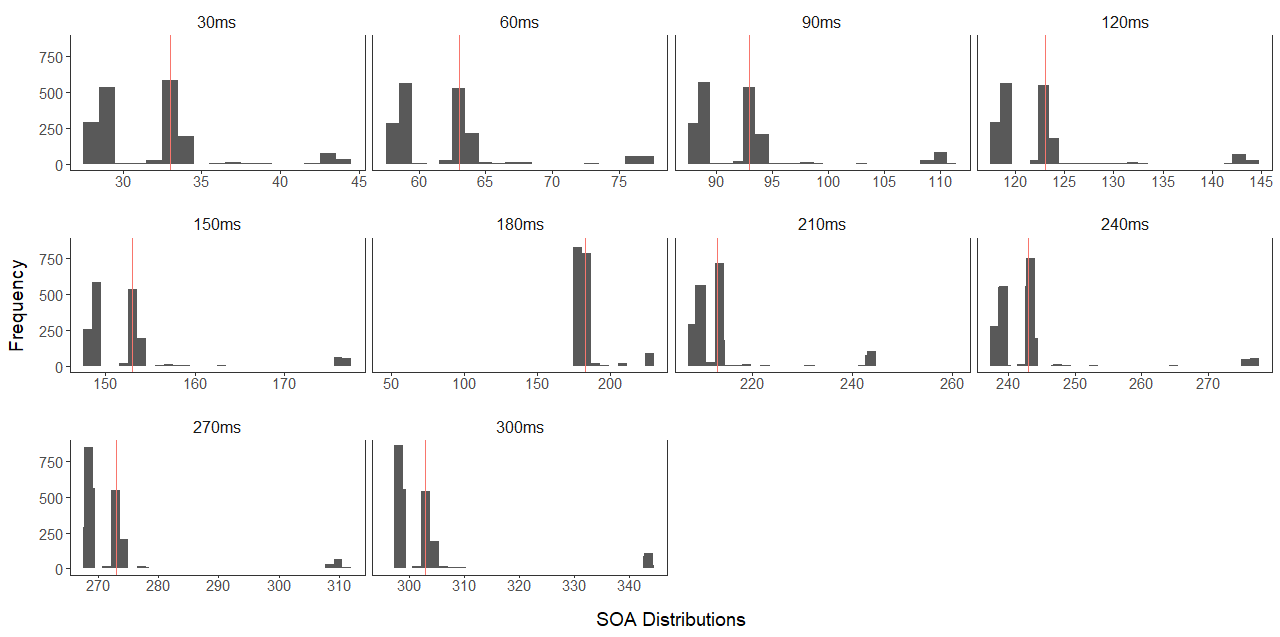


**Figure S2.** The outcome of sensitivity analysis for the interaction effect between cue

validity, threat condition and TMS-SOA. The effect sizes from the actual analysis (Table S1) were used. According to this analysis, the interaction between these three predictors was predicted to be detected with 80% power and alpha of 0.05 with 17 participants.

**
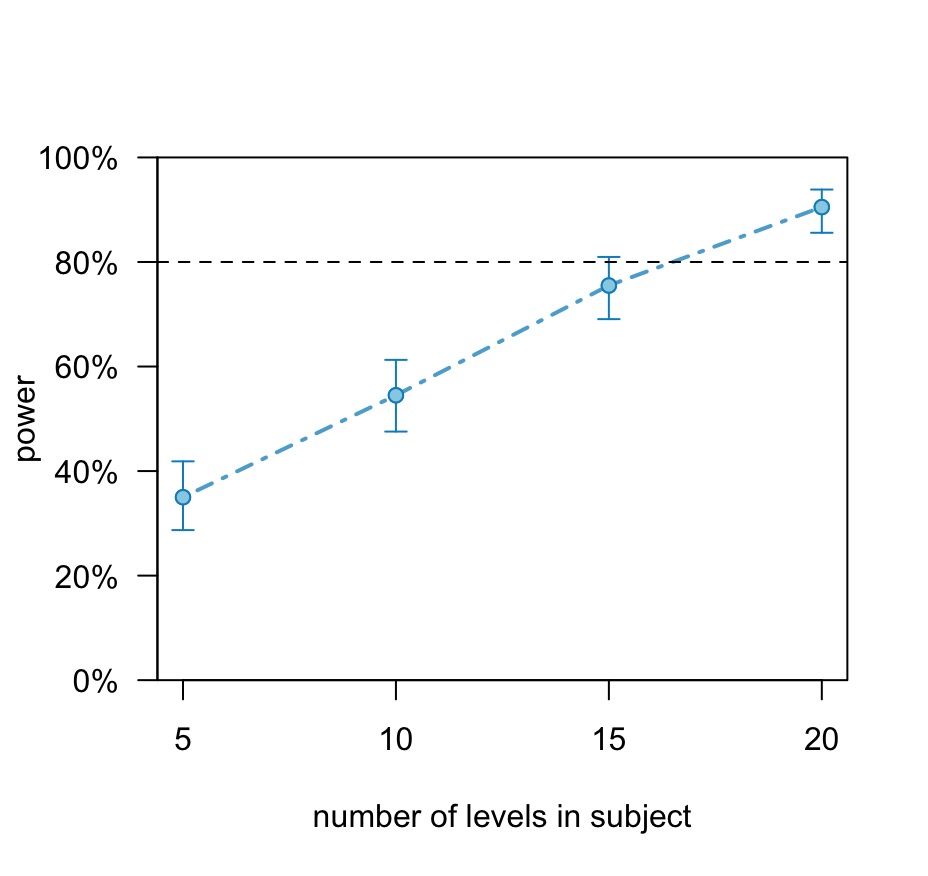
**

**Table S1.** Model testing the effect of TMS pulse at different TMS-SOAs on accuracy. * effects that remain significant following the Bonferroni correction for multiple comparison (α adjusted = .05/6 tests = .0083)

Accuracy

|  | **χ^2^**(df) | ***p*** |
| --- | --- | --- |
| Cue validity | 252.68(1) | <.001* |
| Threat condition | 0.03(1) | .855 |
| TMS SOA | 6.58(9) | .680 |
| Session | 49.37(1) | <.001* |
| Cue validity x Threat | 0.11(1) | .736 |
| Cue validity x TMS SOA | 8.16(9) | .518 |
| Threat condition x TMS SOA | 8.82(9) | .454 |
| Cue validity x Threat condition x TMS SOA | 8.21(9) | .513 |

**Table S2.** Model testing for the effect STAI- trait on accuracy. * effects that remain significant following the Bonferroni correction for multiple comparison (α adjusted = .05/7 tests = . 0071)

|  | **χ^2^**(df) | ***p*** |
| --- | --- | --- |
| Cue validity | 254.04(1) | <.001* |
| Threat condition | 0.04(1) | .844 |
| TMS SOA | 6.25(9) | .714 |
| STAI trait (centered) | 0.28(1) | .596 |
| Session | 49.90(1) | <.001* |
| Cue validity x Threat condition | 0.062(1) | .803 |
| Cue validity x TMS SOA | 7.73(9) | .562 |
| Threat condition x TMS SOA | 8.28(9) | .506 |
| Cue validity x STAI trait | 0.43(1) | .510 |
| Threat condition x STAI trait | 0.48(1) | .486 |
| TMS SOA x STAI trait | 9.04(9) | .433 |
| Cue validity x Threat condition x TMS SOA | 8.31(9) | .503 |
| Cue validity x Threat condition x STAI trait | 0.0007(1) | .978 |
| Cue validity x TMS SOA x STAI trait | 9.89(9) | .359 |
| Threat condition x TMS SOA x STAI trait | 11.21(9) | .261 |
| Cue validity x Threat condition x TMS SOA x STAI trait | 4.48(9) | .877 |

**Table S3.** Time-window of interest analysis for a TMS-SOAs.

|  | TMS-SOA: 60ms – 150 ms | | TMS-SOAs: 180ms-270ms | |
| --- | --- | --- | --- | --- |
|  | **χ^2^**(df) | ***p*** | **χ^2^**(df) | ***p*** |
| Cue validity | 91.45(1) | <.001 | 106.89(1) | <.001 |
| Threat condition | 0.17(1) | .678 | 0.54(1) | .462 |
| TMS SOA | 1.32(1) | .724 | 4.27(1) | .234 |
| Session | 21.11(1) | <.001 | 22.91(1) | <.001 |
| Cue validity x Threat | 0.0005(1) | .982 | 0.16(1) | .688 |
| Cue validity x TMS SOA | 2.81(3) | .421 | 2.92(3) | .404 |
| Threat condition x TMS SOA | 1.15(3) | .765 | 5.19(3) | .158 |
| Cue validity x Threat condition x TMS SOA | 1.85 (3) | .604 | 4.35(3) | .226 |

**Table S4.** Model testing for the effect of target location (left, right) on accuracy. * Effects that remain significant following the Bonferroni correction for multiple comparison (α adjusted = .05/7 tests = .0071)

Accuracy

|  | **χ^2^**(df) | ***p*** |
| --- | --- | --- |
| Cue validity | 255.73(1) | <.001* |
| Threat condition | 0.002(1) | .968 |
| TMS SOA | 7.42(9) | .594 |
| Target position | 29.37(1) | <.001* |
| Session | 49.16(1) | <.001* |
| Cue validity x Threat condition | 0.13(1) | .719 |
| Cue validity x TMS SOA | 9.82(9) | .364 |
| Threat condition x TMS SOA | 9..47(9) | .395 |
| Cue validity x Target position | 0.19(1) | .656 |
| Threat condition x Target position | 0.88(1) | .349 |
| TMS SOA x Target position | 13.27(9) | .151 |
| Cue validity x Threat condition x TMS SOA | 7.73(9) | .561 |
| Cue validity x Threat condition x Target position | 0.47(1) | .494 |
| Cue validity x TMS SOA x Target position | 9.46(9) | .396 |
| Threat condition x TMS SOA x Target position | 7.53(9) | .582 |
| Cue validity x Threat condition x TMS SOA x Target position | 3.44(9) | .944 |

**Table S5.** Model testing accuracy for targets presented specifically in the left visual field and for invalid condition.

Accuracy

|  | **χ^2^**(df) | ***p*** |
| --- | --- | --- |
| Threat condition | 0.63(1) | .425 |
| TMS SOA | 2.99(9) | .965 |
| Session | 21.21(1) | <.001 |
| Threat condition x TMS SOA | 6.58(1) | .681 |

**Table S6.** Model testing the general effect of no-TMS vs. TMS (averaged across all TMS-SOAs) on accuracy. * Effects that remain significant following the Bonferroni correction for multiple comparison (α adjusted = .05/7 tests = .0071)

Accuracy

|  | **χ^2^**(df) | ***p*** |
| --- | --- | --- |
| Cue validity | 72.29(1) | <.001* |
| Threat condition | 3.16(1) | .075 |
| TMS (present vs. absent) | 4.76(1) | .029 |
| Session | 51.95(1) | <.001* |
| Cue validity x Threat | 1.98(1) | .158 |
| Cue validity x TMS | 2.51(1) | .112 |
| Threat condition x TMS | 3.66(1) | .055 |
| Cue validity x Threat condition x TMS | 2.69(1) | .100 |

**Table S7.** Model testing the general effect of no-TMS vs. TMS (averaged across all TMS-SOAs) on reaction time (RT).

RT

|  | **χ^2^**(df) | ***p*** |
| --- | --- | --- |
| Cue validity | 67.93(1) | <.001 |
| Threat condition | 2.67(1) | .10 |
| TMS (present vs. absent) | 2.61(1) | .106 |
| Session | 260.39(1) | <.001 |
| Cue validity x Threat | 1.09(1) | .295 |
| Cue validity x TMS | 0.08(1) | .774 |
| Threat condition x TMS | 1.19(1) | .274 |
| Cue validity x Threat condition x TMS | 1.09(1) | .295 |

**Table S8.** Model testing the effect of RT on accuracy. * Effects that remain significant following the Bonferroni correction for multiple comparison (α adjusted = .05/7 tests = .0071).

Accuracy

|  | Model with TMS present vs absent | | Model with TMS-SOA | |
| --- | --- | --- | --- | --- |
|  | **χ^2^**(df) | ***p*** | **χ^2^**(df) | ***p*** |
| TMS | 6.19(1) | .012 | 6.98(9) | .639 |
| Log RT (centered) | 260.46(1) | <.001* | 536.02(1) | <.001* |
| Cue validity | 59.22(1) | <.001* | 218.12(1) | <.001* |
| Threat condition | 2.37(1) | .123 | 0.51(1) | .476 |
| Session | 15.09(1) | <.001* | 15.90(1) | <.001* |
| TMS x log RT (centered) | 0.44(1) | .508 | 15.62(9) | .075 |
| TMS x cue validity | 2.01(1) | .156 | 10.03(9) | .35 |
| Log RT x cue validity | 14.20(1) | <.001 | 30.32(1) | <.001* |
| TMS x threat condition | 4.15(1) | .041 | 5.74(9) | .765 |
| Log RT x threat condition | 0.27(1) | .599 | 1.53(1) | .215 |
| Cue validity x threat condition | 0.44(1) | .507 | 0.20(1) | .651 |
| TMS x log RT x cue validity | 0.11(1) | .739 | 13.79(9) | .130 |
| TMS x log RT x threat condition | 1.98(1) | .159 | 14.81(9) | .096 |
| TMS x cue validity x threat condition | 1.13(1) | .286 | 6.84(9) | .654 |
| Log RT x cue validity x threat condition | 1.07(1) | .300 | 0.42(1) | .516 |
| TMS x log RT x cue validity x threat condition | 0.37(1) | .538 | 7.946(9) | .539 |

**Table S9.** Model testing the effect of TMS pulse at different TMS-SOAs on the effects of threat and cue validity on accuracy. Model without excluding trials showing high TMS-SOA variability.

Accuracy

|  | **χ^2^**(df) | ***p*** |
| --- | --- | --- |
| Cue validity | 261.28(1) | <.001 |
| Threat condition | 0.007(9) | .935 |
| TMS SOA | 4.65(1) | .864 |
| Session | 32.40(1) | <.001 |
| Cue validity x Threat condition | 2.57(1) | .109 |
| Cue validity x TMS SOA | 7.96(9) | .538 |
| Threat condition x TMS SOA | 6.42(9) | .697 |
| Cue validity x Threat condition x TMS SOA | 7.14(9) | .623 |

**Table S10.** Accuracy as a function of trial number for a model testing TMS SOA. * Effects that remain significant following the Bonferroni correction for multiple comparison (α adjusted = .05/7 tests = .0071).

Accuracy

|  | **χ^2^**(df) | ***p*** |
| --- | --- | --- |
| Cue validity | 253.55(1) | <.001* |
| Threat condition | 0.001(1) | .97 |
| TMS SOA | 7.08(9) | .63 |
| Trial number (centered) | 2.77(1) | .1 |
| Cue validity x Threat condition | .09(1) | .77 |
| Cue validity x TMS SOA | 8.43(9) | .49 |
| Threat condition x TMS SOA | 9.73(9) | .37 |
| Cue validity x Trial number | 3.24(1) | .07 |
| Threat condition x Trial number | .11(1) | .74 |
| TMS SOA x Trial number | 4.72(9) | .86 |
| Cue validity x Threat condition x TMS SOA | 8.96(9) | .44 |
| Cue validity x Threat condition x Trial number | 1.22(1) | .27 |
| Cue validity x TMS SOA x Trial number | 6.78(9) | .66 |
| Threat condition x TMS SOA x Trial number | 8.62(9) | .47 |
| Cue validity x Threat condition x TMS SOA x Trial number | 21.14(9) | .01 |

**Table S11.** A number of trials for each condition included in the final analysis.

|  | Threat (CS+) | | Safe (CS-) | |
| --- | --- | --- | --- | --- |
| TMS-SOA | Cue valid | Cue invalid | Cue valid | Cue invalid |
| 0 | 415 | 428 | 418 | 425 |
| 1 | 344 | 339 | 345 | 340 |
| 2 | 335 | 342 | 334 | 342 |
| 3 | 349 | 338 | 331 | 337 |
| 4 | 346 | 337 | 347 | 352 |
| 5 | 330 | 339 | 337 | 343 |
| 6 | 340 | 327 | 350 | 341 |
| 7 | 339 | 341 | 332 | 345 |
| 8 | 336 | 346 | 341 | 324 |
| 9 | 352 | 333 | 332 | 337 |
| 10 | 334 | 346 | 336 | 343 |

**Table S12.** Model testing the effect of TMS SOA and target location on RT.

RT

|  | **χ^2^**(df) | ***p*** |
| --- | --- | --- |
| TMS SOA | 38.47(9) | <.001 |
| Target position | 1.07(1) | .300 |
| Cue validity | 143.62(1) | <.001 |
| Threat condition | 0.67(1) | .412 |
| Session | 243.85(1) | <.001 |
| TMS-SOA x Target position | 11.52(9) | .241 |
| TMS-SOA x Cue validity | 3.82(9) | .922 |
| Target position x Cue validity | 0.92(1) | .336 |
| TMS-SOA x Threat condition | 10.08(9) | .344 |
| Target position x Threat condition | 1.00(1) | .317 |
| Cue validity x Threat condition | 0.005(1) | .944 |
| TMS-SOA x Target position x Cue validity | 3.62(9) | .935 |
| TMS-SOA x Target position x Threat condition | 4.48(9) | .877 |
| TMS-SOA x Cue validity x Threat condition | 9.59(9) | .385 |
| Target position x Cue validity x Threat condition | 2.96(1) | .085 |
| TMS-SOA x Target position x Cue validity x Threat condition | 11.78(9) | .225 |
